# Synuclein Motor Dysfunction Composite Scale for the Discrimination of Dementia With Lewy Bodies From Alzheimer’s Disease

**DOI:** 10.3389/fnagi.2022.920591

**Published:** 2022-05-19

**Authors:** Ying-Tsung Chen, Satoshi Orimo, Cheng-Yu Wei, Guang-Uei Hung, Shieh-Yueh Yang, Pai-Yi Chiu

**Affiliations:** ^1^Department of Psychiatry, Show Chwan Memorial Hospital, Changhua, Taiwan; ^2^Department of Psychiatry, Chang Bing Show Chwan Memorial Hospital, Changhua, Taiwan; ^3^Department of Neurology, Kamiyoga Setagaya Street Clinic, Tokyo, Japan; ^4^Department of Exercise and Health Promotion, College of Education, Chinese Culture University, Taipei, Taiwan; ^5^Department of Nuclear Medicine, Chang Bing Show Chwan Memorial Hospital, Changhua, Taiwan; ^6^MagQu Co., Ltd., New Taipei, Taiwan; ^7^Department of Neurology, Show Chwan Memorial Hospital, Changhua, Taiwan; ^8^Department of Applied Mathematics, Tunghai University, Taichung, Taiwan

**Keywords:** Synuclein Motor Dysfunction Composite Scale, plasma α-synuclein, dementia with Lewy bodies, Alzheimer’s disease, Motor Dysfunction Questionnaire

## Abstract

**Background:**

An abnormal increase of α-synuclein in the brain is the hallmark of dementia with Lewy bodies (DLB). However, the diagnostic power of plasma α-synuclein in DLB is not yet confirmed. Parkinsonism is highly associated with and is one of the core clinical features of DLB. We studied plasma α-synuclein and developed a novel tool that combined plasma α-synuclein level and Motor Dysfunction Questionnaire (MDQ), namely Synuclein Motor Dysfunction Composite Scale (SMDCS), for the clinical discrimination of DLB from Alzheimer’s disease (AD).

**Methods:**

This cross-sectional study analyzed participants’ demographical data, plasma α-synuclein level, MDQ, structured clinical history questionnaire, neuropsychological and motor function tests, and neuroimaging studies. The power of plasma α-synuclein level, MDQ, and SMDCS for discriminating DLB from non-demented controls (NC) or AD were compared.

**Results:**

Overall, 121 participants diagnosed as 58 DLB, 31 AD, and 31 NC were enrolled. Patients with DLB had significantly higher mean plasma α-synuclein level (0.24 ± 0.32 pg/ml) compared to the NC group (0.08 ± 0.05 pg/ml) and the AD group (0.08 ± 0.05 pg/ml). The DLB group demonstrated higher MDQ (2.95 ± 1.60) compared to the NC (0.42 ± 0.98) or AD (0.44 ± 0.99) groups. The sensitivity/specificity of plasma α-synuclein level, MDQ, and SMDCS for differentiating DLB from non-DLB were 0.80/0.64, 0.83/0.89, and 0.88/0.93, respectively.

**Conclusion:**

Both plasma α-synuclein and MDQ were significantly higher in patients with DLB compared to the NC or AD groups. The novel SMDCS, significantly improved accuracy for the clinical differentiation of DLB from AD or NC.

## Introduction

Dementia with Lewy bodies (DLB) is the second most common degenerative dementia (Geser et al., 2005; Zupancic et al., 2011). An abnormal increase in α-synuclein in the brain is the hallmark of Lewy body disease including Parkinson’s disease (PD) and DLB (Surguchov, 2022). Evidence of biofluid markers for the diagnosis of PD or DLB is still lacking consensus (Li et al., 2007; Noguchi-Shinohara et al., 2009; Ohrfelt et al., 2009; Spies et al., 2009; Kasuga et al., 2010; Hansson et al., 2014; Lin et al., 2017, 2020). Among these studies, the examination of α-synuclein level in the cerebrospinal fluid (CSF) for the differentiation of DLB from other brain disorders has revealed controversial findings. Some studies have shown no difference (Noguchi-Shinohara et al., 2009; Spies et al., 2009) or decreased levels (Kasuga et al., 2010), while others have demonstrated increased levels (Ohrfelt et al., 2009; Hansson et al., 2014). Recent studies have used serum or plasma α-synuclein for the clinical diagnosis of PD or DLB. However, the practicality and feasibility are still being tested (Li et al., 2007; Laske et al., 2011; Lin et al., 2017, 2020). Discrepancies in plasma or serum studies may be due to the level of α-synuclein stored in red blood cells being high, and hemolysis during sample preparation and processing may confound the quantification of α-synuclein (Barbour et al., 2008; Kasuga et al., 2012). Being a novel biomarker, plasma α-synuclein is seldom studied in patients with DLB. To our knowledge, there is only one published study on this topic (Laske et al., 2011). Unlike Alzheimer’s Disease (AD) which uses amyloid and phosphorylated tau in CSF or PET imaging to establish a reliable diagnosis, the determinant imaging or liquid biomarkers in CSF and blood samples for the diagnosis of DLB remains unclear. Hence, more evidence is needed for clinical practice.

Clinical characteristic features including motor and non-motor dysfunctions are still essential for the diagnosis of PD, PD with dementia (PDD), or DLB. In this study, we aimed to test the diagnostic power of plasma α-synuclein for the identification of DLB from AD and non-demented control (NC). In addition, we tested the diagnostic efficacy with our newly developed motor dysfunction questionnaire (MDQ) (Table A1) which has been validated with dopamine transporter imaging by comparing motor dysfunctions among DLB, AD, and ND groups (Chiu et al., 2021). Furthermore, we followed the consensus criteria of using both clinical features and biomarkers for the diagnosis of DLB and hypothesized that a simple diagnostic tool, combining both clinical and biomarker information, should improve the detection of DLB. Therefore, we designed a novel, simple, and practical tool which combined a fluid biomarker (plasma α-synuclein level) and a clinical assessment (MDQ), namely, the Synuclein Motor Dysfunction Composite Scale (SMDCS), for the diagnosis of DLB.

## Materials and Methods

### Participants

This was a cross-sectional study. From 2018 to 2020, a consecutive series of NC, AD, and DLB patients were enrolled from two dementia clinics in central Taiwan. For the diagnosis of NC, the individual must have a global Clinical Dementia Rating Scale (CDR) (Morris, 1993) score of 0 OR 0.5 and without significantly impaired activities of daily living (ADL) defined by a total Instrumental ADL score >6 (Lawton and Brody, 1969). Patients with AD were diagnosed according to the criteria for probable AD developed by the National Institute on Aging and the Alzheimer’s Association workgroup (NIA-AA) (McKhann et al., 2011). Patients with DLB were diagnosed according to the revised consensus criteria for probable DLB, developed by the fourth report of the DLB consortium (McKeith et al., 2017).

### Preparation of Plasma Samples

A 9-ml K3-EDTA tube was used for blood collection. No fasting was required before for blood sampling. The tube was gently inverted several times immediately after blood collection. A swing-out (bucket) rotor was used to centrifuge the blood at room temperature, at 1,500–2,500 × *g*, for 15 min. Aliquots of 0.5-ml plasma were transferred into fresh 2.0-ml tubes. All the aliquoted plasma samples were stored at −80°C within 3 h of blood collection, before the ImmunoMagnetic Reduction (IMR) assays were performed. Plasma samples were collected in 2018–2019 and assayed with IMR (MF-ASC-0060, MagQu) in 2018–2019. The frozen samples are taken directly by MagQu’s staff or delivered in dry ice to MagQu Co., Ltd. for IMR measurements. Each reported concentration of a biomarker was the average of duplicated measurements. The IMR analyzer (XacPro-S) was used for assays.

### Procedures

All participants were selected from Show Chwan Healthcare Center Dementia Registry database (Chiu et al., 2020; Wang et al., 2020; Zhu et al., 2020). The demographical, clinical, neuropsychological, neuropsychiatric, neuroimaging, and laboratory data were collected. The global severity of dementia was assessed according to the CDR scale and the sum of boxes of CDR (CDR-SB). Cognitive functions were assessed with the Cognitive Abilities Screening Instrument (CASI) (Teng et al., 1994) and the Montreal Cognitive Assessment (MoCA) (Nasreddine et al., 2005). Motor functions were assessed with the motor score of the Unified Parkinson’s Disease Rating Scale (UPDRS Part III) (Ballard et al., 1997) and MDQ (Chiu et al., 2021). The novel SMDCS is the sum of the MDQ score and the α-synuclein score. The α-synuclein scores were divided into four according to quartile of the α-synuclein level, from low to high, with a score of 1 representing the lowest quartile and 4 the highest. Neuropsychiatric symptoms were assessed with the 12-domain Neuropsychiatric Inventory (NPI) (Cummings et al., 1994). All patients had results from the following measures: plasma α-synuclein, at least a cerebral CT or MRI, a set of blood screening tests including complete blood count, GOT, GPT, BUN, creatinine, thyroid function, RPR or VDRL, vitamin B12, and folic acid. Furthermore, 96 participants underwent the APOE genetic study, and 82 participants underwent dopamine transporter uptake imaging with ^99m^Tc-Trodat-1 SPECT. Abnormal dopamine transporter imaging (DaTabN) is one of the indicative biomarkers for the diagnosis of DLB.

### Data Analysis

The Chinese version of SPSS for Windows, version 22.0 (IBM, SPSS Inc., Chicago, IL, United States) was used for statistical analyses. Comparisons among the three groups (demographic data, plasma α-synuclein, MDQ, SMDCS, neuropsychological tests, neuropsychiatric symptoms, and UPDRS Part III) were performed with one-way ANOVA. Sex, CDR, APOE4 allele, and clinical features were analyzed using the Chi-square test. The receiver operating characteristic (ROC) curves analysis of plasma α-synuclein, MDQ, and SMDCS were compared between DLB versus non-DLB. Plasma α-synuclein level greater than the cut-off score was defined as α-syn+ group. Dopamine transporter imaging, motor, and non-motor features of SMDCS+ group were compared to SMDCS− group, with odds ratios (OR) adjusted for age and dementia severity by CDR. All calculated *p*-values were two-tailed. Statistical significance was defined as a *p*-value of <0.05.

### Ethical Consideration

The Committee for Medical Research Ethics of Show Chwan Memorial Hospital reviewed the study, and the Data Inspectorate approved it. All participants signed informed consent before participating in the study.

## Results

A total of 121 participants were analyzed in this study, including 59 probable DLB, 31 probable AD, and 31 NC. Comparing the demographical data of the three groups showed that both DLB and AD groups had significantly older age, lower education, higher CDR-SB, lower total CASI score, lower total MoCA score than NC (all *p* < 0.001) (Table 1). Compared to the NC and AD groups, patients with DLB also had significantly higher UPDRS Part III, SBR, and frequencies of all clinical features, including fluctuation of cognition, parkinsonism, visual hallucinations and REM sleep behavior disorder (RBD), and total NPI score (all *p*-value < 0.001). APOE4 allele was significantly higher in patients with AD (58.1%) compared to NC (25.8%) or patients with DLB (32.4%), with a *p*-value of 0.022 (Table 1).

**TABLE 1:** Demographic and background characteristics of NC (*n* = 31), AD (*n* = 31), and DLB (*n* = 59) patients.

	NC	AD	DLB	*F*/χ^2^	*p*-Value	*Post hoc*/pair comparison
Age, mean (SD)	68.3 (9.1)	74.1 (8.3)	77.8 (6.6)	15.41	<0.001	NC < AD < DLB
CDR-SB, mean (SD)	0.5 (0.7)	4.9 (3.7)	5.8 (3.8)	24.96	<0.001	NC < AD < DLB
Female, *n* (%)	15 (48.4)	18 (58.1)	28 (47.5)	0.98	NS	NC = AD = DLB
Education, mean (SD)	8.3 (4.0)	6.3 (4.4)	5.7 (5.3)	3.33	0.039	NC > AD = DLB
IADL, mean (SD)	7.8 (0.5)	4.3 (2.9)	3.6 (2.8)	31.18	<0.001	NC > AD > DLB
MoCA, mean (SD)	21.2 (5.9)	11.7 (6.1)	10.6 (6.1)	32.30	<0.001	NC < AD = DLB
CASI, mean (SD)	84.7 (10.7)	59.5 (21.7)	57.5 (18.7)	25.29	<0.001	NC > AD = DLB
NPI, mean (SD)	3.5 (4.4)	7.0 (11.2)	14.6 (16.3)	8.36	<0.001	NC < AD < DLB
UPDRS-M, mean (SD)	7.1 (5.4)	8.9 (11.3)	21.8 (13.8)	8.36	< 0.001	NC = AD < DLB
VH, *n* (%)	1 (3.2)	2 (6.5)	29 (49.2)	30.60	<0.001	NC = AD < DLB
Parkinsonism, *n* (%)	2 (6.5)	3 (9.7)	49 (83.1)	68.86	<0.001	NC = AD < DLB
RBD, *n* (%)	3 (9.7)	5 (16.1)	34 (57.6)	26.97	<0.001	NC = AD < DLB
DaTabN, *n* (%)	3 (13.6)	4 (26.7)	32 (71.1)	22.78	<0.001	NC = AD < DLB
SBR, mean (SD)	1.71 (0.37)	1.59 (0.44)	1.28 (0.39)	22.78	<0.001	NC > AD > DLB
APOE4, *n* (%)	8 (25.8)	17 (58.1)	11 (32.4)	7.66	0.022	NC = DLB < AD

*n, number of cases; NC, non-demented control; AD, Alzheimer’s Disease; DLB, dementia with Lewy bodies; NA, not applicable; NS, non-significance; CDR-SB, sum of boxes of the Clinical Dementia Rating Scale; α-syn, plasma α-synuclein; HAI-MDQ, Motor Dysfunction Questionnaire in the History-based Artificial Intelligent Clinical Dementia Diagnostic System (HAICDDS), SMDCS: Synuclein Motor Dysfunction Composite Scale; IADL, Instrumental Activities of Daily Living; MoCA, Montreal Cognitive Assessment; CASI, Cognitive Abilities Screening Instrument; NPI, Neuropsychiatric Inventory; UPDRS-M, motor subscale of the Unified Parkinson’s Disease Rating Scale; VH, visual hallucinations; RBD, REM sleep behavior disorder; DaTabN, abnormal dopamine transporter imaging in 22 ND, 15 AD, and 45 DLB; SBR, striatal background ratio; APOE4, apolipoprotein E4 allele in 31 ND, 31 AD, and 34 DLB.*

Patients with DLB had significantly higher mean plasma α-synuclein level (0.24 ± 0.32 pg/ml) compared to NC (0.08 ± 0.05 pg/ml) or patients with AD (0.08 ± 0.05 pg/ml). The DLB group also demonstrated higher MDQ score (3.25 ± 1.77) compared to the NC (0.52 ± 1.15) and AD (0.48 ± 1.06) groups. The SMDCS scores were divided according to the cut-off score of 0.085 pg/ml. Therefore, level 0–0.0424, 0.0425–0.0849, 0.0850–0.1274, and >0.1275 pg/ml were scored 0, 1, 2, and 3, respectively. The SMDCS revealed a higher total score in patients with DLB (4.73 ± 1.84) compared to that of the NC (0.97 ± 1.17) and AD (0.90 ± 1.06) groups (Table 2).

**TABLE 2:** Comparison of plasma α-synuclein, HAI-MDQ, and SMDCS among NC (*n* = 31), AD (*n* = 31), and DLB (*n* = 59) patients.

	NC	AD	DLB	*F*/χ^2^	*p*-Value	*Post hoc*
α-Syn, mean (SD)	0.08 (0.05)	0.08 (0.05)	0.24 (0.32)	7.12	0.001	NC = AD < DLB
HAI-MDQ, mean (SD)	0.52 (1.15)	0.48 (1.06)	3.25 (1.77)	53.13	<0.001	NC = AD < DLB
SMDCS, mean (SD)	0.97 (1.17)	0.90 (1.16)	4.73 (1.84)	91.89	<0.001	NC = AD < DLB

*n, number of cases; NC, non-demented control; AD, Alzheimer’s Disease; DLB, dementia with Lewy bodies; α-syn, plasma α-synuclein; HAI-MDQ, Motor Dysfunction Questionnaire in the History-based Artificial Intelligent Clinical Dementia Diagnostic System (HAICDDS); SMDCS, Synuclein Motor Dysfunction Composite Scale.*

**FIGURE 1:**
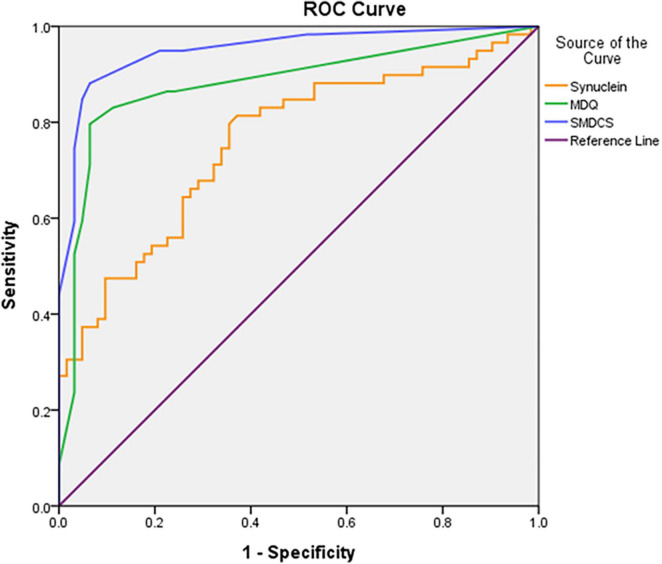
The ROC curves analysis of plasma α-synuclein, MDQ, and SMDCS between DLB versus non-DLB.

Figure 1 demonstrated the ROC curves analysis of plasma α-synuclein, MDQ, and SMDCS between DLB versus non-DLB. The plasma α-synuclein level differentiated patients with DLB from the non-DLB group, with a cut-off score of >0.085, a sensitivity 0.80, a specificity 0.64, and AUC with 95% CI 0.76 (0.67–0.85). The MDQ differentiated patients with DLB from those in the non-DLB group, with a cut-off score of >1, a sensitivity 0.83, a specificity 0.89, and AUC with 95% CI 0.88 (0.81–0.95). The SMDCS significantly differentiated patients with DLB from the non-DLB group, with a cut-off score of >2, a sensitivity 0.88, a specificity 0.93, and AUC with 95% CI 0.95 (0.91–0.99).

Table 3 shows the background characteristics and clinical DLB features of the SMDCS+ (SMDCS > 2) group compared to the SMDCS− (SMDCS ≤ 2) group among all patients, and the OR adjusted for age and dementia severity by CDR-SB. The comparison of demographical data after adjustment showed that the SMDCS+ group had significantly higher plasma α-synuclein level (OR = 20674.16; *p* < 0.001), higher MDQ (OR = 5.56; *p* < 0.001), higher NPI composite score (OR = 1.05; *p* = 0.045), higher DaTabN (OR = 6.84; *p* < 0.001) with lower SBR (OR = 0.22; *p* = 0.028), higher UPDRS Part III (OR = 1.08; *p* = 0.002), and lower education (OR = 0.99; *p* = 0.004). Moreover, all motor and non-motor DLB features were significantly higher in the SMDCS+ group (Table 3).

**TABLE 3:** Dopamine transporter imaging, motor, and non-motor features of SMDCS + group (*n* = 56) compared to SMDCS – group (*n* = 65), odds ratio (OR) adjusted for age and dementia severity by CDR.

	SMDCS+, *n* (%)	SMDCS−, *n* (%)	OR	*p*
α-Syn, mean (SD)	0.24 (0.33)	0.09 (0.05)	20,674.16	<0.001
MDQ, mean (SD)	3.31 (1.33)	0.23 (0.49)	12.01	<0.001
DaTabN, *n* (%)	31 (70.5)	8 (21.1)	7.65	<0.001
SBR, mean (SD)	1.28 (0.41)	1.64 (0.38)	0.11	0.005
UPDRS-M, mean (SD)	22.8 (14.9)	8.2 (7.1)	1.12	<0.001
**Motor features of DLB**				
Resting tremor, *n* (%)	24 (42.9)	4 (6.2)	13.43	<0.001
Kinetic tremor, *n* (%)	31 (55.4)	4 (6.2)	28.43	<0.001
Bradykinesia, *n* (%)	47 (83.9)	6 (9.2)	40.48	<0.001
Rigidity, *n* (%)	42 (75.0)	1 (0.0)	170.58	<0.001
Postural instability	42 (75.0)	3 (4.6)	42.95	<0.001
Parkinsonism*, *n* (%)	50 (89.3)	1 (1.5)	496.02	<0.001
**Non-motor features of DLB**				
Fluctuation, *n* (%)	34 (60.7)	10 (15.4)	4.26	0.003
VHs, *n* (%)	24 (42.9)	8 (12.3)	2.92	0.001
RBD, *n* (%)	32 (57.1)	10 (15.4)	8.07	<0.001

n, number of cases; CDR, Clinical Dementia Rating Scale; OR, odds ratio; α-syn, plasma α-synuclein; MDQ, Motor Dysfunction Questionnaire in the History-based Artificial Intelligent Clinical Dementia Diagnostic System (HAICDDS); DaTabN, abnormal dopamine transporter imaging among 22 ND, 15 AD, and 45 DLB; SBR, striatal background ratio in dopamine transporter imaging among 22 ND, 15 AD, and 45 DLB; Parkinsonism*, bradykinesia plus at least one other parkinsonian motor symptom/sign; UPDRS-M, motor subscale of the Unified Parkinson’s Disease Rating Scale (UPDRS); Fluctuation, fluctuation of cognition; VHs, visual hallucinations; RBD, REM sleep behavior disorder; NS, non-significance.

## Discussion

For the clinical diagnosis of DLB, this study prospectively investigated the plasma α-synuclein level (a fluid biomarker) along with a newly designed MDQ (a clinical assessment). Both tools demonstrated higher scores in patients with DLB compared with the non-DLB group. The combination of both diagnostic tools successfully provided a better discrimination power of the diagnosis of DLB, from AD or NC. In this study, plasma samples were collected and assayed with the IMR standardized procedure. The finding of higher plasma α-synuclein levels in patients with DLB compared to the non-DLB group was not consistent with a 2011 study by Laske et al. (2011). However, the elevated plasma α-synuclein levels concurred with similar studies that used IMR to differentiate PD from non-PD (Lin et al., 2017, 2020). In Laske’s study, they measured α-synuclein serum concentrations using a commercial ELISA kit. Thus, the discrepancy between our study and Laske’s study is probably due to the different preparation methods used for the detection of very low concentrations of α-synuclein in the serum or plasma.

Other important findings in this study also deserved attention. First, demographical data revealed that the participants with DLB in this study had significantly greater motor dysfunctions, including more Parkinsonism and higher UPDRS Part III scores. Besides, patients with DLB also presented higher non-motor features including fluctuation of cognition, VH, and RBD. These findings were compatible with previous studies and the clinical criteria for the diagnosis of DLB (McKeith et al., 2017). In the demographic section of the study, we included other biomarkers which showed higher DaTabN and lower APOE4 allele in patients with DLB compared to those with AD. These findings are also consistent with previous studies.

Second, although a fluid biomarker for the diagnosis of DLB is too novel to become the only indicative or supportive biomarker, findings in our analysis showing that significantly increased levels of plasma α-synuclein in patients with DLB compared to non-DLB patients were evident. This supports the use of a fluid biomarker for the clinical diagnosis of DLB. Findings of significantly higher UPDRS Part III scores in the α-syn + group than in the α-syn – group (18.9 ± 15.7 versus 12.7 ± 14.3; *p* = 0.028) indicated a good correlation between motor dysfunctions and UPDRS and the plasma α-synuclein level. Significantly lower SBR in the α-syn + group than that in the α-syn – group (1.35 ± 0.42 versus 1.55 ± 0.41; *p* = 0.047), and a high negative correlation coefficient of plasma α-synuclein level with SBR (−0.36; *p* = 0.002) indicated a good correlation of the plasma α-synuclein level with reduced dopamine transporter uptake in the striatal areas, which is currently the hallmark of brain imaging study for the diagnosis of DLB. Further studies on fluid biomarkers are warranted for clinical applications.

We assumed that the high rates with different manifestations of Parkinsonian symptoms in the patients with DLB would be noticed by their caregivers. Hence, we used a simple informant-based Parkinsonism questionnaire (MDQ) in this study. We demonstrated that higher MDQ scores (2.95 ± 1.60) among patients with DLB, and much lower scores in the NC (0.42 ± 0.98) and AD (0.44 ± 0.99) groups. These findings revealed that using the MDQ much higher characteristic motor symptoms are observed in patients with DLB compared to non-DLB patients. This finding is compatible with the results of one of our recently published articles (Chiu et al., 2021). Findings of significant higher MDQ in the α-syn + group than in the α-syn – group (2.2 ± 2.0 versus 1.3 ± 1.9; *p* = 0.023) indicated a good correlation between motor dysfunctions as assessed by MDQ and plasma α-synuclein.

Using either the plasma α-synuclein level (sensitivity: 0.80, specificity: 0.64, and AUC: 0.76) or the HAI-MDQ (sensitivity: 0.83, specificity: 0.89, and AUC: 0.88) to discriminate DLB from non-DLB yielded satisfactory results. However, a combination of both of these tools (SMDCS) further increased the power of discrimination (sensitivity of 0.88, specificity of 0.93, and AUC of 0.95). Therefore, we are looking forward to collecting more data and performing analyses on combined complex clinical data, fluid biomarkers, and neuroimaging markers, supplemented with artificial intelligence and machine learning procedures. So that we can provide a more accurate and efficient diagnostic tool for the clinical detection of DLB.

Third, the associated factors of positive SMDCS in all participants in this study had provided the clinical evidence supporting the value of measuring plasma α-synuclein level combined with a clinical questionnaire for the clinical discrimination of DLB from non-DLB. Findings of much higher SMDCS total scores in the SMDCS + group (4.7 ± 1.8) than that in the SMDCS – group (0.9 ± 1.2) and higher UPDRS Part III scores in the SMDCS + group (22.8 ± 14.9) than in the SMDCS – group (8.2 ± 7.1) indicated a good positive correlation of motor dysfunctions between both tools for the assessment of characteristic Parkinsonism associated with DLB. The correlation coefficient of SMDCS with UPDRS Part III was 0.63 in the later analysis. In other words, the DLB motor features are well detected by using a combined scale of both clinical and biofluid markers. Significantly lower SBR in the SMDCS + group (1.28 ± 0.41) than that in the SMDCS – group (1.64 ± 0.38), and a high negative correlation coefficient of SMDCS with SBR (−0.45) indicated a good correlation of the novel tool with reducing dopamine transporter uptake in the striatal areas, which is currently the hallmark of brain imaging study for the diagnosis of DLB. Higher rates of the Parkinsonian symptoms including resting tremor, kinetic tremor, bradykinesia, rigidity, and instability as well as non-motor DLB features including DaTabN, RBD, VH, and cognitive fluctuation were found in the SMDCS+ group revealed that the composite questionnaire for the clinical detection of DLB was simple, practical, and reliable.

## Conclusion

In conclusion, our study showed that both the plasma α-synuclein level and the MDQ score were significantly higher in patients with DLB compared to the NC and AD groups, with a fair diagnostic power. The novel SMDCS which combined both measures is simple and practical. Moreover, the SMDCS demonstrated satisfactory sensitivity and specificity for the clinical differentiation of DLB from AD or NC. The diagnostic value of the novel tool was further confirmed by good correlations with the DAT study and motor subscores of the UPDRS. This simple screening tool can be applied at the bedside, as well as in the clinics, for the screening of motor dysfunctions related to DLB. This could help non-specialists detect DLB more easily in healthcare settings without access to neurologists.

The study has some limitations. First, the sample size in this study was relatively small and included only two hospitals in central Taiwan. Further research studies with larger sample populations for further validation of the findings are needed. Second, the diagnosis of NC, AD, or DLB was based only on the clinical criteria. However, detailed clinical information and DAT imaging may help differentiate DLB from NC or AD. In this study, APOE4 allele was significantly higher in the AD group compared to the DLB and NC groups. Third, the comparison of the plasma α-synuclein level among DLB, AD, and ND was cross-sectional. Therefore, the possible contribution of plasma α-synuclein to the progression of diseases was not able to be speculated in this study. The follow-up of a larger cohort with further observations of the incidence or the progression of dementia should be considered.

## Data Availability Statement

The original contributions presented in the study are included in the article/supplementary material, further inquiries can be directed to the corresponding author.

## Ethics Statement

The studies involving human participants were reviewed and approved by the Show Chwan Memorial Hospital. The ethics committee waived the requirement of written informed consent for participation.

## Author Contributions

Y-TC undertook the literature search and data analysis, edited the author contributions, contributed to revisions of the manuscript, and the final draft of the manuscript. P-YC undertook the literature search and data analysis, contributed to revisions of the manuscript, and the final draft of the manuscript. SO, G-UH, and S-YY contributed to revisions and drafts of the manuscript. All authors contributed to the article and approved the submitted version.

## Conflict of Interest

S-YY was employed by the MagQu Co., Ltd. The remaining authors declare that the research was conducted in the absence of any commercial or financial relationships that could be construed as a potential conflict of interest.

## Publisher’s Note

All claims expressed in this article are solely those of the authors and do not necessarily represent those of their affiliated organizations, or those of the publisher, the editors and the reviewers. Any product that may be evaluated in this article, or claim that may be made by its manufacturer, is not guaranteed or endorsed by the publisher.

## Appendix

**TABLE A1:** Composition of the HAICDDS-Motor Dysfunction Questionnaire (HAI-MDQ). 患者是否有下列症狀？ (不清楚或是不確定請選否) Does the patient have the following symptoms? (If uncertain, please answer No.)

Item			
MD1	坐著或靜止不動的時候，肢體或臉部會明顯地顫抖嗎?	□不會	□會
	Does tremor, or shaking, often in hands, arms, or legs, occur when he/she is sitting or standing still?	□ No	□ Yes
MD2	拿東西，做動作或是說話的時候，肢體或臉部會明顯地顫抖嗎?	□不會	□會
	Does tremor, or shaking, often in hands, arms, or legs, occur when he/she is reaching something, holding position, or talking?	□ No	□ Yes
MD3	動作變得緩慢，尤其是起步特別困難，面部表情也明顯減少嗎?	□不會	□會
	Does movement become slower when he/she try to move from a resting position, and also decreases facial expression?	□ No	□ Yes
MD4	動作變得僵硬，手腳彎曲的角度受到限制，走路步伐變小身體會前傾嗎?	□不會	□會
	Does limited angle or stiff movement occur during moving, the steps become smaller and the posture become stooped?	□ No	□ Yes
MD5	動作變得不穩，走路不平衡，有時候好像要跌倒或真的跌倒嗎?	□不會	□會
	Does balance or posture problems occur during walking and cause frequent falls?	□ No	□ Yes
MD6	若有上述這些動作障礙，剛開始的時候就常常跌倒?	□不會	□會
	Did he/she fall often at the beginning if he/she has these motor dysfunctions mentioned above?	□ No	□ Yes
MD7	說話音調變得平淡，比較沒有抑揚頓挫，會越講越小聲嗎?	□不會	□會
	Does the speech reduce pitch range (monotone) and volume (hypophonia)?	□ No	□ Yes
